# Identification and validation of a major chromosome region for high grain number per spike under meiotic stage water stress in wheat (*Triticum aestivum* L.)

**DOI:** 10.1371/journal.pone.0194075

**Published:** 2018-03-08

**Authors:** Ifeyinwa Onyemaobi, Habtamu Ayalew, Hui Liu, Kadambot H. M. Siddique, Guijun Yan

**Affiliations:** UWA School of Agriculture and Environment and The UWA Institute of Agriculture, The University of Western Australia, Perth WA, Australia; University of Tasmania, AUSTRALIA

## Abstract

Grain number is a major trait for wheat yield under dryland farming. An International Triticeae Mapping Initiative (ITMI) mapping population comprising 105 recombinant inbred lines (RIL) developed from a cross between a Synthetic hexaploid wheat (*Triticum aestivum*) ‘W7984’ and a spring wheat variety ‘Opata M85’ was used to identify quantitative trait loci (QTL) associated with grain number per spike under two treatment conditions, normal watering and water stress during meiosis. Two major QTL for grain number per spike on the main stem *Q*.*Gnu*.*uwa-5A-1* and *Q*.*Gnu*.*uwa-5A-2* with phenotypic variations of 25.71% and 24.93%, respectively, were detected on the long arm of chromosome 5A when plants were exposed to water stress during meiosis. One QTL (*Q*.*Gnu*.*uwa-2A*) with a LOD score of 2.8 was detected on the long arm of chromosome 2A under normal watering condition. The alleles associated with higher grain number per spike under different treatment conditions came from the Synthetic W7984 parent. Two populations developed from crosses Synthetic W7984 × Lang and Synthetic W7984 × Westonia were used to validate the identified QTL under water stress during meiosis. SSR markers Xbarc230 and Xbarc319 linked with the identified QTL on chromosome 5AL were validated in the two F_2:4_ segregating populations. These closely linked SSR markers could potentially be utilized in marker-assisted selection to reduce yield loss in regions where water stress during meiosis occurs frequently. The identified QTL can be incorporated into elite lines / cultivars to improve wheat grain yield.

## Introduction

Bread wheat (*Triticum aestivum* L.) is one of the most important food crops consumed by humans [1]. It is grown on more than 220 million hectares of land worldwide and provides about 20% of the global daily requirements for calories and proteins [2]. One of the primary aims of plant breeding projects is to improve crop yield to ensure food security for the ever-increasing world population [3], especially under adverse environmental conditions, such as dryland farming that generally leads to significant reductions in grain yield.

Grain yield is a complex trait that integrates many components and developmental processes, with gene expressions that are often strongly influenced by the environment [4]. Grain number per spike (GN) and thousand grain weight (TGW) are two of the main components of grain yield in wheat [5]. The study of individual yield components would provide better insight into the genetics of plant development and how it affects yield performance [6,7]. At physiological maturity, final grain number determines grain yield in wheat, such that yield increases have been directly associated with increases in grain number [8–12].

Water stress is a frequent abiotic stress under rainfed agriculture, which can significantly reduce grain yield [1]. For example, water stress reduced wheat yield by 1.1 t/ha during the 2006/2007 cropping season in Australia, resulting in 46% yield reduction when compared to the previous (2005/2007 cropping season) year record [13]. Water stress resistance can be defined as the ability of plants to survive and produce measurable yield under periodic water stress or limited water supply [14,15]. Therefore, it is essential to breed crop varieties that are resistant to water stress and maintain yield under dryland farming.

In Mediterranean-type environments, crops often experience water stress during the reproductive or grain-filling phases [16,17]. Meiosis is a brief, yet unique stage during the reproductive processes in plants, and a short duration of water stress during this growth phase disrupts grain yield. Water stress during meiosis results in fewer grains and consequently lower grain yield [10,18,19]. Recent studies have indicated that yield reductions due to water stress during meiosis in wheat arises from the loss of viability of both male and female reproductive parts without any significant reduction in grain weight [19–21].

Grain number is a quantitative trait, and quantitative trait loci (QTL) analysis is an important tool for determining the chromosomal regions or locating genes underlying its genetic variation [22]. QTL studies will facilitate a better understanding of the relationship between grain number and yield. Extensive studies have been conducted to identify the genetic basis of grain number in diploid crops such as rice, maize, tomato and barley [23,24]. For example, the loss of function of the gene *OsCKX2* in rice contributes to higher grain number [23,25].

QTL clusters for yield and yield components have been identified on different chromosomes scattered or clustered across the wheat genome [26–31]. S1 Table shows a list of different yield-related QTL. Abiotic stress that coincides with the meiotic process can greatly reduce grain number. However, most yield-trait related analyses have focused more on the identification, validation and/or cloning of genes associated with grain weight while few studies have focused on the identification of gene(s) or QTL influencing grain number in wheat, specifically under water stress treatment during meiosis [32–36]. Identification of these chromosome regions/gene(s) associated with grain number would enhance the efficiency of selection and breeding of new wheat varieties with improved water stress resistance.

Our research focused on the genetic control and molecular basis of grain number per spike in wheat plants exposed to water stress during meiosis. The objectives of this study were to identify genome regions controlling grain number per spike under water stress during meiosis, validate the identified chromosome regions using molecular markers in different mapping populations and identify suitable wheat grain number-associated markers for marker assisted selection (MAS).

## Materials and methods

### Plant materials

A recombinant inbred line (RIL) mapping population developed from a cross between the Synthetic W7984 (Altar84/*Aegilops tauschii* (219) CIGM86.940, female) and Opata (Opata M85, male) [37] along with the parents was used in this QTL identification study. A total of 105 RIL lines were evaluated with seeds obtained from the Australian Grains Genebank. Two F_2:4_ populations, Synthetic W7984 × Westonia, and Synthetic W7984 × Lang were generated and used for QTL validation and detection of the chromosomal segments from the donor parent (Synthetic W7984) into different progenies. Seventy lines from each validation population were randomly selected for validation. The closely associated SSR markers to the identified QTL were used to genotype segregating lines from the crosses mentioned above.

### Plant growth and treatment

Plants were grown in polyvinyl chloride pots (9 cm diameter, 37 cm height), in a controlled-temperature glasshouse where day and night temperatures were maintained at 22±2°C and 15±2°C, respectively at The University of Western Australia (31° 57ʹ S, 115° 47ʹ E) from April to September 2015. Each pot, with a 9-mm hole at the bottom to allow free drainage of water was filled with 2.1 kg of sterilised soil mixture (5: 2: 3 fine composted pine barks: coco peat: brown river sand, pH ~ 6.0). Three seeds were sown per pot for each RIL and later thinned to one plant per pot after seven days. A randomized complete block design with three replications and two treatments − normal watering (control) and water stress was used, where the locations of the pots with respect to genotype, replicate and treatment were random within the blocks. Scotts Peters^®^ Excel^®^ water soluble nutrient fertilizer with 15% nitrogen (11.6% as nitrate nitrogen, 1.4% as ammoniacal nitrogen, 2.0% as urea nitrogen), 2.2% phosphorus (soluble in neutral ammonium citrate and water), 12.4% potassium (as potassium nitrate), 5.0% calcium (as calcium nitrate), 1.8% magnesium (as magnesium nitrate), 0.12% iron, 0.06% manganese, 0.02% boron, 0.015% copper, 0.015% zinc, and 0.010% molybdenum was supplied weekly from 21 days after sowing. Nutrient fertilizer was not applied during the treatment period. For the measurement of field (pot) capacity of the soil media, four free draining pots, each containing 2.1 kg of sterilised soil mixture, were flooded with water and allowed to drain for 48h. Two samples from each pot were taken, and their fresh weight and dry weight were measured using a balance before and after oven-drying, respectively. The per cent water content of the soil mixture at field capacity was calculated as 26% (w/w), using the following formula:

$$\%\;\mathrm{soil}\;\mathrm{water}\;\mathrm{content}=\frac{FW-DW}{DW}\times 100$$

where, FW = fresh weight, and DW = dry weight of the samples. Each pot was weighed daily to record the mass of water loss by evapotranspiration. By the next watering the minimum field capacity of the pots was around 60%. Based on the amount of moisture lost, each pot was watered to bring it up to 80% field capacity. When the auricle distance (AD)—the distance between the auricles of the flag leaf and the second last leaf of the plants—reached 0 cm, water stress was imposed by withholding water entirely for 7 days [20]. Wheat grain yield per plant is determined mainly by the contribution of the main stem under rainfed conditions [38]. The stress treatment of each pot was based on the AD of the main stem of each plant to monitor the effect of water stress on the total grain number on the main stem, which hereafter is referred to as grain number per spike. Soil water content measurements were conducted to determine plant water status during the stress treatment. During the imposed water stress treatment, the pots were weighed daily to monitor the field capacity but were not watered throughout the 7 days stress period. However, watering was resumed in the stress group as per the normal watering group after the stress treatment. Field capacity at the end of the stress period was between 40% - 45%. The daily pot weighing was used to record the amount of water loss, corresponding to the daily transpiration of the plants or water use during the stress period. The field capacity was maintained at about 80% for the whole plant life apart from the stress period until the stage of physiological maturity when watering was stopped.

### QTL analysis

The Synthetic W7984 × Opata M85 molecular linkage map constructed by [37] was used. The map was downloaded from the GrainGenes website (https://wheat.pw.usda.gov/cgi-bin/GG3/report.cgi?class=image;name=Wheat,+Synthetic+x+Opata,+BARC+markers,+5A, accessed 10 May 2017). The map had a total of 1,475 SSR and RFLP markers distributed across the 21 linkage groups. To ensure maximum genome coverage and reduce errors due to missing values, 1,017 markers were selected from the linkage analysis with an average marker density of 1 cM after filtering for 60% missing values. The locations and effects of QTL were determined following the composite interval mapping method (CIM). Windows QTL (WinQTL) Cartographer v2.5 software was used to perform CIM analysis [39]. The CIM analysis was run using a backward stepwise regression method of a window size of 10 cM and a step size of 1 cM. The significant threshold LOD scores for QTL detection were determined based on 1,000 permutations at P ≤ 0.05 [40]. The logarithm of odds (LOD) peak location ≥ 2.5 was used to declare a QTL for both water stress and normal growing conditions. For cM position, a 0 cM position indicates the first (most distal) marker on the short arm of the chromosome. The confidence intervals for the QTL was determined by locating the markers on both sides of the QTL peak that correspond to a decrease in 1 LOD score relative to the peak marker [41]. Only SSR markers within the 1-LOD support interval was used for validating the identified QTL. Adjacent QTL on the same chromosome for the same trait was considered as different when the intervals between them was not overlapping.

### DNA isolation and PCR

Genomic DNA was extracted from the leaves of three-week-old seedlings of individual plants from the parental lines of Synthetic W7984, Westonia and Lang, and each of the F_2:4_ populations (70 lines from Synthetic W7984 × Lang and 70 lines from Synthetic W7984 × Westonia) using a modified CTAB method [42,43]. A NanoDrop ND-1000 (Thermo Fisher Scientific) was used to measure the quality and quantity of the total DNA samples. The primers were obtained from Sigma-Aldrich (Sigma-Aldrich Pty Ltd, NSW, Australia). Polymerase chain reaction (PCR) contained 50 ng genomic DNA as template, 1× Bioline MyTaq™ reaction buffer (containing 1mM dNTPs, 3mM MgCl_2_, stabilizers and enhancers), 0.20 μM of forward and reverse primers and 1 U MyTaq™ DNA Polymerase (Bioline, NSW, Australia) in a total volume of 15 μL. The PCR reactions were conducted using an EppendorfMaster cycler ep Gradient S thermocycler (Eppendorf, NY, USA) programmed at: 94°C for 5 mins, 35 cycles of denaturation at 94°C for 30 s, annealing at varying temperatures obtained from GrainGenes for the different selected SSR markers for 30 s, elongation at 72°C for 45 s, and a final extension at 72°C for 5 mins. The primers were obtained from Sigma-Aldrich (Sigma-Aldrich Pty Ltd, NSW, Australia).

PCR was run for each marker and their products were analysed based on a previously described method by [44] using a LabChip® GX Touch 24 (PerkinElmer, Massachusettes, USA).

### Statistical data analysis

The main stems of the parents and each RIL were tagged, and at full physiological maturity, grain number per spike was evaluated from the tagged stem only. The populations (F_2:4_) used for the validation studies were F_2:4_ lines (derived from selfed F_2_ single seed descent method). These lines were also evaluated similarly. The mean values for the control and water-stressed treatments were used for QTL analysis. As expected, the numbers of heterozygotes among the F_2:4_ lines screened were few, hence they were excluded from the analysis. Only F_2:4_ plants homozygous for the Synthetic W7984 marker allele and those homozygous for the Westonia/Lang marker allele were used to validate the identified QTL. They were placed into two separate allele groups: lines that were homozygous for Synthetic W7984 marker allele (group 1) and lines with Westonia or Lang marker allele (group 2) for the different SSR marker and validation population. Phenotypic statistical data analysis was conducted using Genstat statistical software 17^th^ edition [45]. Analysis of variance was conducted based on the following fixed effects model: Y_ij_ = μ + g_j_ + ε_ij_ where Y_ij_ is observed mean, μ is population mean; g_j_ is an effect due to the jth genotype, and ɛ_ij_ is random error. Heritability analysis was conducted using the formula: $h^{2}=\delta_{g}^{2}/(\delta_{g}^{2}+\delta_{e}^{2})$ where $\delta_{g}^{2}\;and\;\delta_{e}^{2}$ are the estimated genotypic and error variances, respectively [46]. The genotypic and error variances were estimated as: $\delta_{g}^{2}=\frac{MSg-MSe}{r}$ and $\delta_{e}^{2}=\frac{MSe}{r}$ where MSg is mean square of the RILs, MSe is the residual error, and r is the number of replicates.

## Results

### Phenotypic variation of wheat grain number per spike

**Fig 1:**
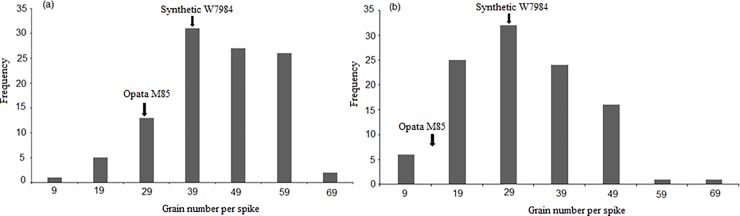
**Frequency distribution of phenotypic variation for wheat grain number per spike among 105 RILs under (a) control (normal watering) and (b) water stress during meiosis.** Grain number per spike for Synthetic W7984 and Opata M85 parental lines under control and water-stressed conditions are indicated by arrows. Values shown are means.

The phenotypic data analysis of variance showed significant (P < 0.01) differences for grain number per spike among the 105 RILs of Synthetic W7984×Opata M85 under both normal growing conditions (control) and water stress during meiosis (Table 1). The mean grain number per spike was 39.7 under control and 26.5 under water stress during meiosis. The frequency distribution of mean grain number per spike in the RIL population under normal growing conditions and under water stress during meiosis are presented in Fig 1. The Synthetic W7984 parent had higher grain numbers per spike under normal and water-stressed conditions than the Opata M85 parent (S2 Table). Broad sense heritability was 51% for grain number per spike under stress and 70% under normal growing conditions (Table 1). The high broad sense heritability indicated that genetic factors strongly influenced grain number per spike variation in the mapping population.

**Table 1:** Mean squares, genotypic and phenotypic coefficients of variation, and broad sense heritability of wheat grain number per spike under water stress during meiosis and normal growth conditions (control).

Grain number	MSg	MSe	$\delta_{g}^{2}$	$\delta_{e}^{2}$	$\delta_{p}^{2}$	H^2^
**Water stress during meiosis**	301.3**	148.0	51.1	49.3	100.4	0.51
**Control**	209.3**	64.5	48.3	21.5	69.8	0.70

MSg: mean square of genotype; MSe: mean square of random error; $\delta_{g}^{2}$: estimated genetic variance; $\delta_{e}^{2}$: estimated error variance; $\delta_{p}^{2}$: estimated phenotypic variance; H^2^: broad sense heritability.

** indicates significant difference at P < 0.01.

### QTL analysis for grain number per spike

**Fig 2:**
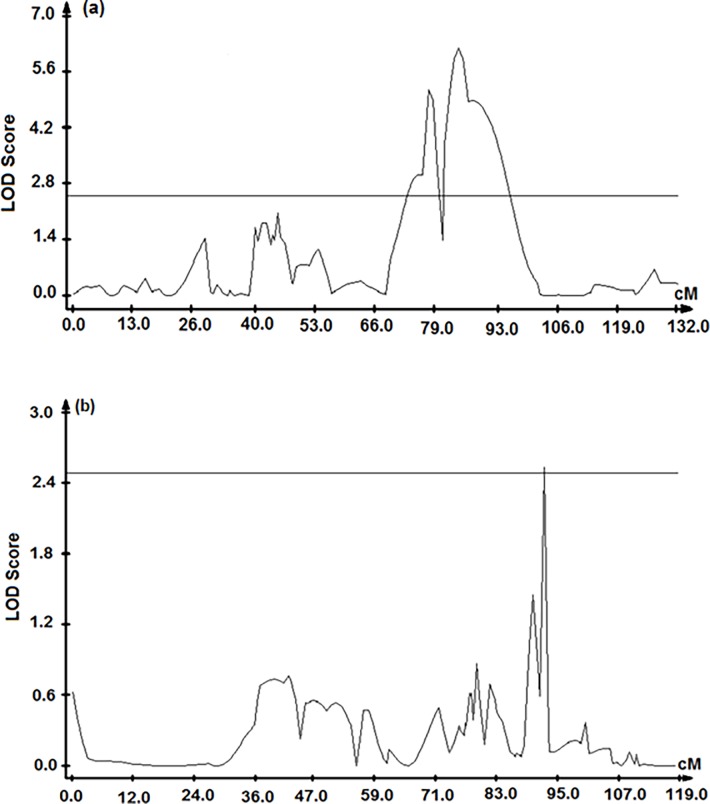
**QTL identified in the Synthetic W7984 × Opata M85 RIL population which were associated with grain number per spike (a) under water stress during meiosis, located on chromosome 5A and (b) under normal watering, located on chromosome 2A using composite interval mapping.** The horizontal bars indicate the significant LOD thresholds for the QTL detection which was set at ≥ 2.5 for both treatments.

QTL for grain number per spike were detected under both normal and water stress during meiosis conditions (Fig 2; Table 2). The only significant QTL for grain number per spike under water stress during meiosis were identified on the long arm of chromosome 5A (*Q*.*Gnu*.*uwa-5A-1* and *Q*.*Gnu*.*uwa-5A-2*) with LOD scores of 5.2 and 6.2 respectively—close to SSR markers Xbarc151, Xbarc230, Xgwm666 and Xbarc319—and explained 25.7% and 24.9% of the phenotypic variation respectively. *Q*.*Gnu*.*uwa-5A-1* and *Q*.*Gnu*.*uwa-5A-2* were mapped 6.5 cM apart. One QTL was detected on chromosome 2A (*Q*.*Gnu*.*uwa-2A*) for grain number per spike under normal watering conditions, which explained 8% of the phenotypic variation and had a LOD score of 2.8. Synthetic W7984 parent contributed high grain number alleles under both normal and water stress during meiosis conditions.

**Table 2:** Genetic characteristics of QTL associated with grain number per spike in the Synthetic W7984 × Opata M85 RIL population. QTL was detected by composite interval mapping. QTL peak position (cM) is based on linkage between markers from the Synthetic W7984 × Opata M85 molecular linkage map constructed by Song et al., 2005. Selected flanking markers include SSR markers on both sides of the QTL peak with 1 LOD score reduction relative to the peak marker. All QTL were detected at LOD ≥ 2.5 threshold following 1,000 permutations, the percent phenotypic variance (R^2^%) and size of the additive effect are also presented.

Treatment	QTL name	QTL peak position (cM)	Flanking SSR markers	LOD score	R^2^ (%)	Additive effect
**Water stress during meiosis**	*Q*.*Gnu*.*uwa-5A-1*	77.7	Xgwm666 and Xbarc230	5.17	25.71	0.61
	*Q*.*Gnu*.*uwa-5A-2*	84.2	Xbarc230 and Xbarc319	6.20	24.93	0.59
**Control**	*Q*.*Gnu*.*uwa-2A*	92.1	Xgwm312 and Xbarc353	2.77	8.04	0.36

### Validation of the QTL for grain number per spike under water stress during meiosis

Four SSR markers—Xbarc151, Xbarc230, Xbarc319 and Xgwm666—were tightly linked with the two identified QTL for grain number per spike under water stress during meiosis. The peak position of the *Q*.*Gnu*.*uwa-5A-1* was 77.7 cM while *Q*.*Gnu*.*uwa-5A-2* was 84.2 cM; the selected four markers span between 64.0 cM and 86.4 cM on wheat chromosome 5A. Only two markers, Xbarc230 and Xbarc319, were polymorphic between the parental lines of Synthetic W7984 × Lang and Synthetic W7984 × Westonia and were therefore used for the QTL validation.

**Fig 3:**
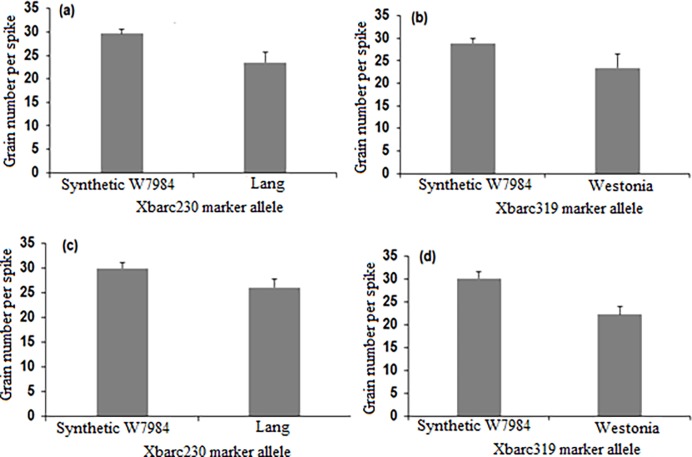
Validation of SSR markers Xbarc230 and Xbarc319 associated with the identified QTL for grain number per spike under water stress during meiosis. (a) and (c) indicate F_2:4_ lines homozygous for Xbarc230 marker alleles from Synthetic W7984 and Lang parental lines, (b) and (d) indicate F_2:4_ lines homozygous for Xbarc319 marker alleles from Synthetic W7984 and Westonia parental lines. Values shown are means and error bars are standard errors of the means. Two-sample t-test revealed that under water stress during meiosis, the mean difference in grain number per spike between the allele combination groups in different F_2:4_ validation populations was statistically significant (P ≤ 0.05) for both Xbarc230 and Xbarc319 marker alleles.

**Fig 4:**
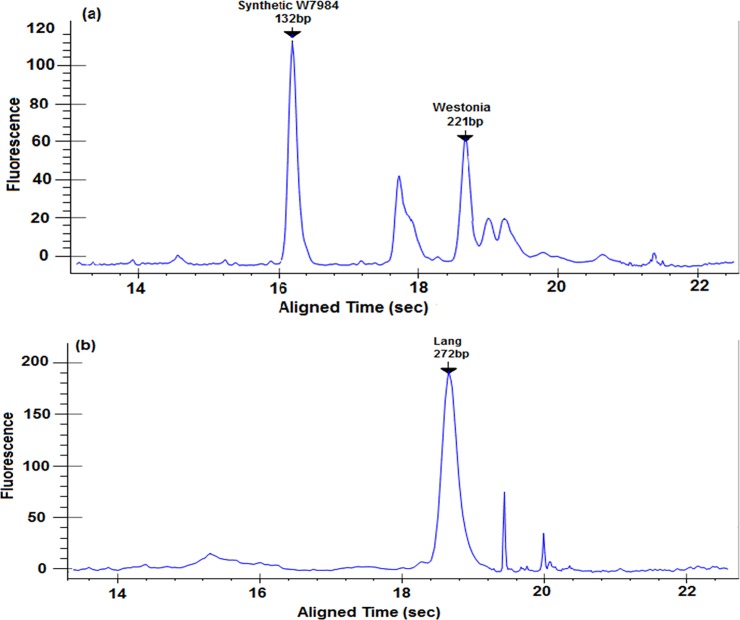
Electropherogram of PCR products of marker Xbarc230 and Xbarc319 from F_2:4_ Synthetic W7984 × Westonia lines exposed to water stress during meiosis. The calculated molecular weight (bp) of PCR product (peaks) is displayed, the black arrows point to amplified fragment sizes (a) indicates an F_2:4_ plant with the alleles of both Synthetic W7984 and Westonia parental lines for SSR marker Xbarc230 (b) indicates an F_2:4_ plant amplified by SSR marker Xbarc319 and possessing the allele of Lang parental line alone.

Plants possessing different alleles of markers Xbarc230 (closest to *Q*.*Gnu*.*uwa-5A-1*) and Xbarc319 (closest to *Q*.*Gnu*.*uwa-5A-2*) were separated into allele group 1 and allele group 2 using LabChip®. The fragment sizes in Table 3 were used to score the randomly selected 70 lines from each of the F_2:4_ populations (Synthetic W7984 × Westonia and Synthetic W7984 × Lang) into different groups based on the allele combination observed for the different genotypes. The mean performance of genotypes based on two types of allele combination groups (1 and 2) were used to calculate the phenotypic effect of the identified QTL under water stress during meiosis. Generally, plants in group 1 had higher grain numbers per spike compared with those in group 2 (Fig 3). Fig 4A and 4B shows the electropherogram of PCR products for F_2:4_ individual plants derived Synthetic W7984 × Westonia amplified by Xbarc230 and Xbarc319, respectively. The two-sample t-test revealed that under water stress during meiosis, the mean difference in grain number per spike between the allele combination groups in the different F_2:4_ validation populations was statistically significant (P ≤ 0.05) for both Xbarc230 and Xbarc319 (Fig 3).

**Table 3:** Fragment size of the two SSR markers, with polymorphism among the parents, associated with the identified QTL for grain number per spike under water stress in three parental lines, Synthetic W7984, Lang and Westonia.

SSR markers	Parental lines	Fragment size (bp)
**Xbarc230**	Synthetic W7984	132
	Lang	257
	Westonia	222
**Xbarc319**	Synthetic W7984	203
	Lang	274
	Westonia	243

## Discussion

The two major QTL identified in this research—*Q*.*Gnu*.*uwa-5A-1* and *Q*.*Gnu*.*uwa-5A-2*—contributing to variations in grain number per spike under water stress during meiosis were both located on the long arm of wheat chromosome 5A, a region known to carry major genes influencing adaptability and productivity [26, 47, 48]. The genomic region harbouring these two closely-liked major QTL explained 25.7% and 24.9% of the total phenotypic variation, respectively. Wheat chromosome 5A is known to carry several gene(s) influencing adaptability and productivity [29, 48–50]. Chromosome 5A also plays a crucial role in drought resistance. For example, Quarrie et al. [51] identified a QTL on chromosome 5A with a major effect on drought-induced ABA accumulation in wheat. Genomic regions and gene(s) of agronomic importance have been mapped on the long arm of wheat chromosome 5A, with examples including genes for reduced vernalisation response (*Vrn-A1*), ear emergence time, spike morphology, and awn development [47,48,52].

Vernalisation, the initiation of flowering by prolonged exposure to cold temperatures, is a major determinant of flowering time. Börner et al [26] already identified flowering time QTL in Synthetic W7984 and Opata M85 RIL mapping population on chromosomes 2D, 3A and 5D. The two major QTL—*Q*.*Gnu*.*uwa-5A-1* and *Q*.*Gnu*.*uwa-5A-2*—contributing to variations in grain number per spike under water stress during meiosis, were found to be located close to the vernalisation gene *Vrn-A1* based on the physical positions (obtained by blasting against the wheat reference genome) of the gene and the identified QTL (https://urgi.versailles.inra.fr). However, the QTL results for final auricle distance (measured at the end of the stress period), number of days from final AD measurement to anthesis, the number of days from sowing to anthesis, tiller number per plant, and water use during the stress period suggested that the 5AL QTL were not due to a staging artefact caused by *Vrn-A1* segregation, because the *Vrn-A1* alleles segregating in the population were not functionally different with regard to flowering time under the experimental conditions.

SSR markers act as anchors in genetic mapping and are potentially useful for MAS [53]. Xbarc230 and Xbarc319 could be potential markers for plant breeding to reduce yield loss in regions where water stress during meiosis occurs often. Whether the identified QTL region in chromosome 5AL contains one or more key genes for water stress resistance during the meiotic process requires further investigation through generating near isogenic lines and fine mapping of *Q*.*Gnu*.*uwa-5A-1* and *Q*.*Gnu*.*uwa-5A-2* with more DNA markers.

QTL for final auricle distance (measured at the end of the stress period), number of days from final AD measurement to anthesis, number of days from sowing to anthesis, tiller number per plant, and water use during the stress period were mapped on almost all the chromosomes except 2A, 3B, 4B and 7B [S3 Table; S4 Table; S5 Table; S6 Table; S7 Table; S8 Table]. None of the identified QTL associated with these developmental traits occurred in the same chromosome region as the identified QTL for grain number per spike on chromosome 5AL (Table 2; S3 Table). However, a region on chromosome 2D contained QTL effects for both number of days from the final AD measurement to anthesis and number of days from sowing to anthesis. By blasting against the wheat reference genome (https://urgi.versailles.inra.fr), it was found that photoperiod sensitivity (*Ppd_D1*) gene is closely located to the identified 2D QTL (*Q*.*Ndan*.*uwa-2D* and *Q*.*Snan*.*uwa-2D*). Therefore, *Ppd_D1* might have influence on the QTL for number of days from the final AD measurement to anthesis and number of days from sowing to anthesis. Chromosome 5AL has also been reported to harbour the most repeatable grain yield QTL across different mapping populations, environments and treatments [30,54,55]. Cuthbert et al. [30] detected two grain yield QTL and eight yield-related QTL on the long arm of chromosome 5A. QTL that control both water stress resistant related traits and yield component traits have been detected in this chromosome region [50]. In this study, we showed that chromosome 5AL affects grain number per spike. Such unique attributes highlight the significant contributions of the long arm of chromosome 5A to yield improvements and some of those QTL may coincide with the QTL detected in this study.

A QTL, *Q*.*Gnu*.*uwa-2A*, relating to grain number per spike under normal water conditions, was detected on chromosome 2AL. SSR markers Xgwm312 and Xbarc353 were linked to the identified QTL. *Q*.*Gnu*.*uwa-2A* is in a similar position to a QTL for grain number per ear reported by Huang et al. [54]. In other studies, QTL associated with yield traits such as relative water content, plant height, days to heading, spikelets per spike and awn length have been detected on chromosome 2A [56,57].

Clusters of yield QTL have also been identified on different wheat chromosomes, either controlling yield itself or a yield component. The results of this study agreed with the report by Peng et al. [56] on the presence of highly significant (P ≤ 0.001) yield-related QTL on both wheat chromosomes 2A and 5A. The favourable allele for grain number per spike under normal growing conditions and water stress during meiosis was contributed by the Synthetic W7984 parent. In the study by Börner et al. [26], two major QTL with LOD > 3.0 and seven minor QTL with LOD between 2 and 3, detected for grain yield and yield-related traits across different growing seasons and environments, all came from the Synthetic W7984 parent. The present finding is consistent with other research that identified drought-tolerant genes and adaptive traits in Synthetic W7984 and other Synthetic wheat lines derived from the diploid wild goat grass *Aegilops tauschii* [58–60], which suggests that the Synthetic hexaploid wheat W7984 could harbour a suite of yield-related adaptive features that should be further explored. Fine mapping of the QTL loci on the long arm of chromosome 5A is now in progress in our laboratory.

Grain number is a quantitative trait influenced by both genetic and environmental factors. Water stress is a yield-limiting factor and the most intense effects on yield have been recorded when stress coincides with the period between the onset of meiosis and early grain initiation [61, 62]. In major wheat-growing areas, particularly those with a Mediterranean climate, water stress during the meiotic process occurs often. Successful completion of meiosis results in viable gamete production which will ensure grain number production. Making direct selections for grain yield components and breeding high-yielding wheat cultivars with high grain numbers will positively influence grain yield [63]. In the present study, identifying grain number per spike QTL under water stress during meiosis and its effect on grain yield portrays how the selection and genetic analysis of a single yield component could be used to potentially increase grain yield. Consequently, the identified and validated favourable alleles could be transferred into wheat cultivars used in dryland farming which often experience water stress during the meiotic process, to make them more tolerant to drought and increase grain yield.

## Supporting information

S1 Table Summary of yield-related QTL identified for water-stress resistance in wheat. (DOCX)

S2 Table Mean grain number per spike for Synthetic W7984 parent, Opata M85 parent and 105 recombinant inbred lines (RILs) of Synthetic W7984×Opata M85 under both normal watering (control) and water stress during meiosis. (DOCX)

S3 Table QTL associated with different developmental traits under water stress during meiosis (S) and under control (C) condition using Synthetic W7984 × Opata M85 RIL population. QTL was detected by composite interval mapping. QTL peak position (cM) is based on linkage between markers from the Synthetic W7984 × Opata M85 molecular linkage map constructed by Song et al., 2005. All QTL were detected at LOD ≥ 2.5 threshold following 1,000 permutations, the percent phenotypic variance (R2%) and the additive allele effect are also presented.
(DOCX)

S4 Table Mean final auricle distance (AD) measurement (cm) for Synthetic W7984 parent, Opata M85 parent and 105 recombinant inbred lines (RILs) of Synthetic W7984×Opata M85 under both normal watering (control) and water stress during meiosis. (DOCX)

S5 Table Mean number of days from final auricle distance (AD) measurement to anthesis for Synthetic W7984 parent, Opata M85 parent and 105 recombinant inbred lines (RILs) of Synthetic W7984×Opata M85 under both normal watering (control) and water stress during meiosis. (DOCX)

S6 Table Mean number of days from sowing to anthesis for Synthetic W7984 parent, Opata M85 parent and 105 recombinant inbred lines (RILs) of Synthetic W7984×Opata M85 under both normal watering (control) and water stress during meiosis. (DOCX)

S7 Table Mean tiller number per plant for Synthetic W7984 parent, Opata M85 parent and 105 recombinant inbred lines (RILs) of Synthetic W7984×Opata M85 under both normal watering (control) and water stress during meiosis. (DOCX)

S8 Table Mean amount of water use (corresponding to the daily transpiration of the pots) which was determined by weighing the pots daily (kg) under water stress treatment during meiosis for Synthetic W7984 parent, Opata M85 parent and 105 recombinant inbred lines (RILs) of Synthetic W7984×Opata M85. (DOCX)
