# Supplementary material for: Identification and validation of a major chromosome region for high grain number per spike under meiotic stage water stress in wheat (Triticum aestivum L.)
Source: PLoS One. 2018 Mar 8;13(3):e0194075. doi: 10.1371/journal.pone.0194075 (PMC5843344; doi:10.1371/journal.pone.0194075)
Supplement: S1 Table — (DOCX) [file pone.0194075.s001.docx]

S1 Table. Summary of yield-related QTL identified for water-stress resistance in wheat.

| **Trait** | **Chromosome location** | **Phenotypic variance (%)** | **Reference** |
| --- | --- | --- | --- |
| Grain yield | 4A | 27% | [1] |
| Grain yield, grain fill rate, grains m^-2^, biomass production, biomass production rate, and drought susceptivity index | 4AL | 20, 33, 23, 30, 26, and 41% respectively | [2] |
| Grain number per ear | 7AL and 7BL | 22 and 35% respectively | [3] |
| Grain yield | 2BL | 21.5% | [4] |
| Grain yield, shoot biomass, plant height, and harvest index | 4B | 27% | [5] |
| Grain yield | 2D | 25.7% | [6] |
| Grain yield and canopy temperature | 3B | 22% | [7] |
| Fertile spikelet number per spike | 1A | 24.1 | [8] |
| Thousand-grain weight | 4A | 21% | [9] |
| Grain yield | 3A | 26.1% | [10] |
| Thousand-grain weight | 4D | 31.8% | [11] |
| Grain number per ear | 1A and 5D | 23.2 and 22.6% respectively | [12] |
| Grain yield | 3B | 21.6% | [13] |
| Thousand-grain weight | 7B.1 and 7B.2 | 20.6 and 25.9% respectively | [13] |

**References**

1. Pinto RS, Pinto M, Reynolds K, Mathews CL, McIntyre J-J, Olivares Villegas S. Heat and drought adaptive QTL in a wheat population designed to minimize confounding agronomic effects. Theor Appl Genet. 2010;121:1001−1021.

2. Kirigwi FM, Van Ginkel M, Brown-Guedira G, Gill BS, Paulsen GM, Fritz AK. Markers associated with a QTL for grain yield in wheat under drought. Mol Breeding. 2007;20:401−413.

3. Quarrie SA, Steed A, Calestani C, Semikhodskii A, Lebreton C, Chinoy C, et al. A high-density genetic map of hexaploid wheat (*Triticum aestivum* L.) from the cross Chinese Spring × SQ1 and its use to compare QTLs for grain yield across a range of environments. Theor Appl Genet. 2005;110:865−880. doi: 10.1007/s00122−004−1902−7

4. Maccaferri M, Sanguineti MC, Corneti S, Ortega JLA, Salem MB, Bort J, et al. Quantitative trait loci for grain yield and adaptation of durum wheat (*Triticum durum* Desf.) across a wide range of water availability. Genetics. 2008;178:489−511.

5. Kadam S, Singh K, Shukla S, Goel S, Vikram P, Pawar V, et al. Genomic associations for drought tolerance on the short arm of wheat chromosome 4B. Funct Integr Genomics. 2012;12:447−464.

6. Verma V, Verma MJ, Foulkes AJ, Worland R, Caligari JW, Caligari JW. Mapping quantitative trait loci for flag leaf senescence as a yield determinant in winter wheat under optimal and drought-stressed environments. Euphytica. 2004;135:255−263.

7. Bennett D, Reynolds M, Mullan D, Izanloo A, Kuchel H, Langridge P, et al. Detection of two major grain yield QTL in bread wheat (*Triticum aestivum* L.) under heat, drought and high yield potential environments. Theor Appl Genet. 2012;125:1473−1485.

8. Xu Y−F, Li S−S, Li L−H, Ma F−F, Fu X−Y, Shi Z−L, et al. QTL mapping for yield and photosynthetic related traits under different water regimes in wheat. Mol Breeding. 2017;37:34. doi: 10.1007/s11032−016−0583−7

9. Nezhad KZ, Weber WE, Roder MS, Sharma S, Lohwasser U, Meyer RC, et al. QTL analysis for thousand-grain weight under terminal drought stress in bread wheat (*Triticum aestivum* L.). Euphytica. 2012;186:127−138. doi: 10.1007/s10681−011−0559−y

10. Campbell BT, Baenziger PS, Gill KS, Eskridge KM, Budak H, Erayman M, et al. Identification of QTLs and environmental interactions associated with agronomic traits on chromosome 3A of wheat research partially funded by USDA, NRICGP 00-353000-9266. Nebraska Agricultural Research Division, Journal Series No. 13824. Crop Sci. 2003;43:1493−1505. doi: 10.2135/cropsci2003.1493

11. McCartney CA, Somers DJ, Humphreys DG, Lukow O, Ames N, Noll J, et al. Mapping quantitative trait loci controlling agronomic traits in the spring wheat cross RL4452×'AC Domain'. Genome. 2005;48:870−883.

12. Hai L, Guo H, Wagner C, Xiao S, Friedt W. Genomic regions for yield and yield parameters in Chinese winter wheat (*Triticum aestivum* L.) genotypes tested under varying environments correspond to QTL in widely different wheat materials. Plant Sci. 2008;175:226−232. doi: http://dx.doi.org/10.1016/j.plantsci.2008.03.006

13. Huang XQ, Cöster H, Ganal MW, Röder MS. Advanced backcross QTL analysis for the identification of quantitative trait loci alleles from wild relatives of wheat (*Triticum aestivum* L.). Theor Appl Genet. 2003;106(8):1379−1389. doi: 10.1007/s00122−002−1179−7
