# Supplementary material for: Identification and validation of a major chromosome region for high grain number per spike under meiotic stage water stress in wheat (Triticum aestivum L.)
Source: PLoS One. 2018 Mar 8;13(3):e0194075. doi: 10.1371/journal.pone.0194075 (PMC5843344; doi:10.1371/journal.pone.0194075)
Supplement: S2 Table — (DOCX) [file pone.0194075.s002.docx]

S2 Table. Mean grain number per spike for Synthetic W7984 parent, Opata M85 parent and 105 recombinant inbred lines (RILs) of Synthetic W7984×Opata M85 under both normal watering (control) and water stress during meiosis.

| **Plant** | **Mean grain no per spike** | | **Plant** | **Mean grain no per spike** | |
| --- | --- | --- | --- | --- | --- |
|  | **Control** | **Water stress** |  | **Control** | **Water stress** |
| Synthetic W7984 | 35.00 | 28.50 | SO_056 | 46.50 | 47.00 |
| Opata M85 | 24.00 | 10.50 | SO_057 | 39.50 | 34.50 |
| SO_001 | 52.50 | 11.50 | SO_058 | 32.50 | 14.50 |
| SO_002 | 45.00 | 34.00 | SO_059 | 41.00 | 34.00 |
| SO_003 | 51.00 | 20.00 | SO_060 | 34.50 | 28.50 |
| SO_004 | 51.50 | 28.00 | SO_061 | 31.50 | 20.50 |
| SO_005 | 51.00 | 46.50 | SO_062 | 50.00 | 32.00 |
| SO_006 | 52.00 | 14.50 | SO_063 | 41.00 | 24.50 |
| SO_007 | 32.50 | 27.00 | SO_064 | 42.50 | 26.50 |
| SO_008 | 51.50 | 47.00 | SO_065 | 28.00 | 19.50 |
| SO_009 | 53.00 | 45.00 | SO_066 | 26.50 | 12.00 |
| SO_010 | 40.50 | 21.50 | SO_067 | 43.00 | 22.50 |
| SO_011 | 26.00 | 11.50 | SO_068 | 40.00 | 17.50 |
| SO_012 | 30.00 | 29.00 | SO_069 | 28.00 | 7.00 |
| SO_014 | 28.00 | 8.50 | SO_071 | 50.00 | 34.00 |
| SO_015 | 53.00 | 14.00 | SO_072 | 35.00 | 20.00 |
| SO_016 | 38.50 | 14.00 | SO_073 | 27.50 | 8.50 |
| SO_017 | 40.50 | 22.00 | SO_074 | 44.00 | 26.00 |
| SO_018 | 49.00 | 48.50 | SO_075 | 48.00 | 22.50 |
| SO_019 | 56.00 | 24.00 | SO_076 | 43.00 | 32.50 |
| SO_020 | 52.00 | 24.50 | SO_077 | 29.00 | 12.00 |
| SO_021 | 50.50 | 31.50 | SO_078 | 38.00 | 20.00 |
| SO_022 | 28.00 | 26.50 | SO_079 | 22.50 | 10.50 |
| SO_023 | 17.00 | 17.00 | SO_080 | 18.00 | 22.50 |
| SO_024 | 50.00 | 45.50 | SO_081 | 32.00 | 9.00 |
| SO_025 | 44.00 | 36.50 | SO_082 | 9.00 | 11.00 |
| SO_026 | 36.00 | 10.00 | SO_083 | 35.00 | 6.50 |
| SO_029 | 57.50 | 39.50 | SO_084 | 17.50 | 11.00 |
| SO_030 | 37.50 | 36.00 | SO_085 | 18.00 | 10.50 |
| SO_031 | 37.00 | 26.50 | SO_086 | 15.50 | 4.00 |
| SO_032 | 46.00 | 20.50 | SO_088 | 38.50 | 19.00 |
| SO_033 | 51.00 | 42.00 | SO_089 | 30.00 | 13.50 |
| SO_034 | 51.00 | 41.00 | SO_090 | 27.00 | 24.00 |
| SO_035 | 39.00 | 30.00 | SO_091 | 38.50 | 22.50 |
| SO_036 | 33.50 | 34.50 | SO_092 | 55.00 | 43.50 |
| SO_037 | 37.00 | 19.00 | SO_093 | 29.50 | 23.00 |
| SO_038 | 54.00 | 34.00 | SO_094 | 22.00 | 12.00 |
| SO_039 | 55.00 | 31.00 | SO_095 | 52.00 | 29.00 |
| SO_040 | 37.00 | 10.50 | SO_096 | 27.50 | 16.00 |
| SO_041 | 49.50 | 36.50 | SO_097 | 33.50 | 13.00 |
| SO_042 | 34.50 | 12.50 | SO_098 | 37.00 | 21.00 |
| SO_043 | 41.00 | 36.50 | SO_099 | 41.00 | 45.50 |
| SO_044 | 58.00 | 24.00 | SO_100 | 37.50 | 29.00 |
| SO_045 | 64.00 | 29.50 | SO_101 | 38.50 | 42.50 |
| SO_046 | 34.50 | 29.50 | SO_102 | 42.00 | 34.50 |
| SO_047 | 45.00 | 37.50 | SO_103 | 42.00 | 42.50 |
| SO_048 | 32.50 | 24.00 | SO_104 | 59.00 | 61.00 |
| SO_049 | 36.00 | 23.50 | SO_106 | 33.50 | 35.50 |
| SO_050 | 60.00 | 43.50 | SO_110 | 54.50 | 60.00 |
| SO_051 | 45.50 | 36.00 | SO_111 | 42.00 | 16.00 |
| SO_052 | 41.50 | 41.00 | SO_112 | 41.00 | 31.00 |
| SO_053 | 49.00 | 33.50 | SO_113 | 29.00 | 18.50 |
| SO_054 | 39.50 | 29.50 | SO_114 | 30.00 | 23.00 |
| SO_055 | 52.50 | 46.00 |  |  |  |
