# Supplementary material for: Identification and validation of a major chromosome region for high grain number per spike under meiotic stage water stress in wheat (Triticum aestivum L.)
Source: PLoS One. 2018 Mar 8;13(3):e0194075. doi: 10.1371/journal.pone.0194075 (PMC5843344; doi:10.1371/journal.pone.0194075)
Supplement: S3 Table — QTL was detected by composite interval mapping. QTL peak position (cM) is based on linkage between markers from the Synthetic W7984 × Opata M85 molecular linkage map constructed by Song et al., 2005. All QTL were detected at LOD ≥ 2.5 threshold following 1,000 permutations, the percent phenotypic variance (R2%) and the additive allele effect are also presented. (DOCX) [file pone.0194075.s003.docx]

S3 Table: QTL associated with different developmental traits under water stress during meiosis (S) and under control (C) condition using Synthetic W7984 × Opata M85 RIL population. QTL was detected by composite interval mapping. QTL peak position (cM) is based on linkage between markers from the Synthetic W7984 × Opata M85 molecular linkage map constructed by Song et al., 2005. All QTL were detected at LOD ≥ 2.5 threshold following 1,000 permutations, the percent phenotypic variance (R^2^ %) and the additive allele effect are also presented.

| **Trait** | **Treatment** | **QTL name** | **QTL peak** | **LOD** | **R^2^ %** | **Additive effect** |
| --- | --- | --- | --- | --- | --- | --- |
| Final AD (Fiad) | S | *Q.Fiad.uwa-1A-1* | 5.11 | 3.42 | 9.01 | 0.53 |
|  |  | *Q.Fiad.uwa-1A-2* | 65.9 | 5.44 | 15.03 | -0.69 |
|  |  | *Q.Fiad.uwa-3A* | 88.3 | 5.07 | 14.95 | 0.77 |
|  |  | *Q.Fiad.uwa-6A* | 2.0 | 2.74 | 6.31 | -0.39 |
|  |  | *Q.Fiad.uwa-7A* | 38.9 | 2.86 | 8.36 | 0.49 |
|  | C | *Q.Fiad.uwa-1B* | 60.1 | 3.72 | 10.63 | 0.41 |
|  |  | *Q.Fiad.uwa-1D* | 104.4 | 2.63 | 7.72 | -0.33 |
|  |  | *Q.Fiad.uwa-5D* | 14.6 | 6.42 | 25.58 | 0.60 |
|  |  | *Q.Fiad.uwa-6D* | 1.0 | 3.16 | 9.75 | 0.37 |
| Days from final AD measurement to anthesis (Ndan) | S | *Q.Ndan.uwa-1B* | 28.9 | 3.43 | 9.01 | 1.39 |
|  |  | *Q.Ndan.uwa-2D* | 20.7 | 6.49 | 22.10 | 2.17 |
|  | C | *Q.Ndan.uwa-2D* | 21.1 | 8.93 | 21.45 | 5.30 |
|  |  | *Q.Ndan.uwa-4D* | 85.5 | 2.72 | 6.54 | 2.84 |
| Days from sowing to anthesis (Snan) | S | *Q.Snan.uwa-2D* | 21.1 | 8.66 | 22.00 | 7.06 |
|  | C | *Q.Snan.uwa-2D* | 27.4 | 2.87 | 9.57 | 1.93 |
|  |  | *Q.Snan.uwa-4A* | 61.9 | 2.63 | 7.64 | -1.76 |
|  |  | *Q.Snan.uwa-6A* | 47.3 | 3.46 | 10.45 | 2.19 |
| Tiller number per plant (Trno) | S | *Q.Trno.uwa-6B* | 10.2 | 2.87 | 9.05 | -0.72 |
|  |  | *Q.Trno.uwa-7A* | 95.8 | 2.71 | 8.40 | 0.73 |
|  | C | *Q.Trno.uwa-2B* | 4.5 | 2.77 | 8.01 | 0.40 |
|  |  | *Q.Trno.uwa-3D* | 67.8 | 3.50 | 10.37 | -0.45 |
|  |  | *Q.Trno.uwa-5B* | 150.3 | 3.89 | 12.73 | 0.50 |
| Water use during stress period (Wusp) | S | *Q.Wusp.uwa-5A* | 11.1 | 3.22 | 11.39 | -0.05 |

The negative and positive signs of additive effects show the origin of the increasing allele: positive sign indicates Synthetic W7984 while negative sign shows Opata M85 as the origin
