# Supplementary material for: Identification and validation of a major chromosome region for high grain number per spike under meiotic stage water stress in wheat (Triticum aestivum L.)
Source: PLoS One. 2018 Mar 8;13(3):e0194075. doi: 10.1371/journal.pone.0194075 (PMC5843344; doi:10.1371/journal.pone.0194075)
Supplement: S4 Table — (DOCX) [file pone.0194075.s004.docx]

S4 Table. Mean final auricle distance (AD) measurement (cm) for Synthetic W7984 parent, Opata M85 parent and 105 recombinant inbred lines (RILs) of Synthetic W7984×Opata M85 under both normal watering (control) and water stress during meiosis.

| **Plant** | **Final AD(cm)** | | **Plant** | **Final AD(cm)** | |
| --- | --- | --- | --- | --- | --- |
|  | **Control** | **Water stress** |  | **Control** | **Water stress** |
| Synthetic W7984 | 11.60 | 12.32 | SO_056 | 11.10 | 4.85 |
| Opata M85 | 10.30 | 9.80 | SO_057 | 11.60 | 9.55 |
| SO_001 | 11.60 | 8.65 | SO_058 | 12.85 | 11.00 |
| SO_002 | 10.50 | 9.80 | SO_059 | 10.45 | 6.65 |
| SO_003 | 10.95 | 7.50 | SO_060 | 10.15 | 6.75 |
| SO_004 | 11.50 | 7.85 | SO_061 | 9.20 | 7.25 |
| SO_005 | 10.90 | 9.10 | SO_062 | 10.50 | 8.40 |
| SO_006 | 12.00 | 7.40 | SO_063 | 12.45 | 11.30 |
| SO_007 | 10.85 | 6.80 | SO_064 | 9.85 | 7.00 |
| SO_008 | 11.00 | 9.05 | SO_065 | 10.65 | 8.65 |
| SO_009 | 10.50 | 7.35 | SO_066 | 10.70 | 7.10 |
| SO_010 | 9.90 | 9.55 | SO_067 | 11.25 | 9.15 |
| SO_011 | 10.40 | 8.15 | SO_068 | 13.35 | 7.90 |
| SO_012 | 10.50 | 8.15 | SO_069 | 10.90 | 11.25 |
| SO_014 | 10.00 | 8.00 | SO_071 | 10.40 | 9.40 |
| SO_015 | 10.50 | 7.85 | SO_072 | 9.95 | 6.65 |
| SO_016 | 10.75 | 8.70 | SO_073 | 8.60 | 8.00 |
| SO_017 | 11.30 | 8.35 | SO_074 | 10.15 | 7.75 |
| SO_018 | 10.50 | 8.40 | SO_075 | 9.70 | 6.80 |
| SO_019 | 12.00 | 6.25 | SO_076 | 11.40 | 7.00 |
| SO_020 | 11.00 | 9.10 | SO_077 | 10.10 | 6.70 |
| SO_021 | 10.00 | 7.15 | SO_078 | 11.35 | 8.45 |
| SO_022 | 11.75 | 9.25 | SO_079 | 11.30 | 8.25 |
| SO_023 | 12.05 | 8.00 | SO_080 | 11.55 | 12.25 |
| SO_024 | 12.00 | 10.15 | SO_081 | 12.25 | 9.50 |
| SO_025 | 11.50 | 7.40 | SO_082 | 12.40 | 7.50 |
| SO_026 | 11.30 | 7.50 | SO_083 | 10.75 | 7.80 |
| SO_029 | 12.30 | 8.65 | SO_084 | 14.00 | 8.40 |
| SO_030 | 10.15 | 6.65 | SO_085 | 11.75 | 6.65 |
| SO_031 | 11.05 | 10.75 | SO_086 | 10.85 | 7.75 |
| SO_032 | 11.80 | 9.00 | SO_088 | 10.90 | 8.64 |
| SO_033 | 13.40 | 9.25 | SO_089 | 9.80 | 8.35 |
| SO_034 | 11.75 | 7.30 | SO_090 | 9.65 | 9.30 |
| SO_035 | 11.40 | 8.65 | SO_091 | 9.85 | 7.40 |
| SO_036 | 9.90 | 7.50 | SO_092 | 14.60 | 10.25 |
| SO_037 | 11.35 | 10.80 | SO_093 | 10.65 | 8.60 |
| SO_038 | 8.85 | 6.90 | SO_094 | 10.75 | 7.70 |
| SO_039 | 11.65 | 8.40 | SO_095 | 10.70 | 9.75 |
| SO_040 | 11.40 | 6.00 | SO_096 | 9.85 | 7.85 |
| SO_041 | 9.00 | 6.70 | SO_097 | 10.85 | 6.80 |
| SO_042 | 10.40 | 7.60 | SO_098 | 11.60 | 9.40 |
| SO_043 | 8.95 | 8.80 | SO_099 | 12.60 | 8.20 |
| SO_044 | 9.60 | 8.70 | SO_100 | 11.50 | 11.00 |
| SO_045 | 9.40 | 6.75 | SO_101 | 10.75 | 8.65 |
| SO_046 | 9.00 | 8.45 | SO_102 | 11.20 | 7.30 |
| SO_047 | 9.85 | 7.60 | SO_103 | 9.00 | 6.65 |
| SO_048 | 8.75 | 6.80 | SO_104 | 10.50 | 7.25 |
| SO_049 | 9.10 | 7.50 | SO_106 | 11.65 | 8.75 |
| SO_050 | 12.25 | 9.40 | SO_110 | 10.40 | 7.65 |
| SO_051 | 9.70 | 8.85 | SO_111 | 13.25 | 10.85 |
| SO_052 | 10.30 | 10.00 | SO_112 | 10.90 | 10.00 |
| SO_053 | 12.20 | 9.80 | SO_113 | 12.40 | 7.25 |
| SO_054 | 11.40 | 8.10 | SO_114 | 10.60 | 9.55 |
| SO_055 | 12.75 | 12.50 |  |  |  |
