# Supplementary material for: Identification and validation of a major chromosome region for high grain number per spike under meiotic stage water stress in wheat (Triticum aestivum L.)
Source: PLoS One. 2018 Mar 8;13(3):e0194075. doi: 10.1371/journal.pone.0194075 (PMC5843344; doi:10.1371/journal.pone.0194075)
Supplement: S5 Table — (DOCX) [file pone.0194075.s005.docx]

S5 Table. Mean number of days from final auricle distance (AD) measurement to anthesis for Synthetic W7984 parent, Opata M85 parent and 105 recombinant inbred lines (RILs) of Synthetic W7984×Opata M85 under both normal watering (control) and water stress during meiosis.

| **Plant** | **No of days from final AD measurement to anthesis** | | **Plant** | **No of days from final AD to measurement to anthesis** | |
| --- | --- | --- | --- | --- | --- |
|  | **Control** | **Water stress** |  | **Control** | **Water stress** |
| Synthetic W7984 | 68 | 50 | SO_056 | 66 | 41 |
| Opata M85 | 76 | 48 | SO_057 | 51 | 34 |
| SO_001 | 71 | 43 | SO_058 | 64 | 39 |
| SO_002 | 51 | 40 | SO_059 | 51 | 37 |
| SO_003 | 51 | 39 | SO_060 | 51 | 38 |
| SO_004 | 51 | 35 | SO_061 | 64 | 45 |
| SO_005 | 76 | 45 | SO_062 | 51 | 38 |
| SO_006 | 72 | 43 | SO_063 | 64 | 39 |
| SO_007 | 54 | 36 | SO_064 | 50 | 44 |
| SO_008 | 53 | 40 | SO_065 | 54 | 34 |
| SO_009 | 50 | 42 | SO_066 | 63 | 39 |
| SO_010 | 52 | 33 | SO_067 | 47 | 40 |
| SO_011 | 68 | 43 | SO_068 | 71 | 46 |
| SO_012 | 51 | 37 | SO_069 | 74 | 45 |
| SO_014 | 54 | 34 | SO_071 | 57 | 37 |
| SO_015 | 82 | 48 | SO_072 | 54 | 36 |
| SO_016 | 73 | 46 | SO_073 | 50 | 40 |
| SO_017 | 72 | 43 | SO_074 | 50 | 43 |
| SO_018 | 51 | 38 | SO_075 | 50 | 44 |
| SO_019 | 55 | 37 | SO_076 | 50 | 44 |
| SO_020 | 70 | 43 | SO_077 | 64 | 39 |
| SO_021 | 60 | 37 | SO_078 | 66 | 42 |
| SO_022 | 52 | 40 | SO_079 | 66 | 42 |
| SO_023 | 56 | 40 | SO_080 | 77 | 46 |
| SO_024 | 69 | 46 | SO_081 | 76 | 45 |
| SO_025 | 51 | 39 | SO_082 | 91 | 53 |
| SO_026 | 60 | 38 | SO_083 | 66 | 44 |
| SO_029 | 50 | 40 | SO_084 | 69 | 42 |
| SO_030 | 55 | 41 | SO_085 | 56 | 39 |
| SO_031 | 78 | 46 | SO_086 | 58 | 36 |
| SO_032 | 51 | 35 | SO_088 | 74 | 44 |
| SO_033 | 53 | 44 | SO_089 | 87 | 51 |
| SO_034 | 51 | 39 | SO_090 | 68 | 46 |
| SO_035 | 73 | 46 | SO_091 | 54 | 39 |
| SO_036 | 70 | 47 | SO_092 | 52 | 33 |
| SO_037 | 85 | 51 | SO_093 | 50 | 40 |
| SO_038 | 62 | 47 | SO_094 | 50 | 43 |
| SO_039 | 73 | 48 | SO_095 | 68 | 41 |
| SO_040 | 76 | 45 | SO_096 | 62 | 38 |
| SO_041 | 51 | 37 | SO_097 | 52 | 33 |
| SO_042 | 62 | 38 | SO_098 | 54 | 39 |
| SO_043 | 50 | 45 | SO_099 | 50 | 42 |
| SO_044 | 51 | 41 | SO_100 | 76 | 45 |
| SO_045 | 50 | 50 | SO_101 | 51 | 33 |
| SO_046 | 77 | 46 | SO_102 | 51 | 38 |
| SO_047 | 51 | 38 | SO_103 | 62 | 38 |
| SO_048 | 48 | 48 | SO_104 | 50 | 48 |
| SO_049 | 55 | 42 | SO_106 | 61 | 38 |
| SO_050 | 65 | 44 | SO_110 | 51 | 37 |
| SO_051 | 65 | 40 | SO_111 | 76 | 45 |
| SO_052 | 51 | 35 | SO_112 | 87 | 51 |
| SO_053 | 51 | 38 | SO_113 | 60 | 43 |
| SO_054 | 66 | 45 | SO_114 | 77 | 48 |
| SO_055 | 69 | 42 |  |  |  |
