# Supplementary material for: Identification and validation of a major chromosome region for high grain number per spike under meiotic stage water stress in wheat (Triticum aestivum L.)
Source: PLoS One. 2018 Mar 8;13(3):e0194075. doi: 10.1371/journal.pone.0194075 (PMC5843344; doi:10.1371/journal.pone.0194075)
Supplement: S6 Table — (DOCX) [file pone.0194075.s006.docx]

S6 Table. Mean number of days from sowing to anthesis for Synthetic W7984 parent, Opata M85 parent and 105 recombinant inbred lines (RILs) of Synthetic W7984×Opata M85 under both normal watering (control) and water stress during meiosis.

| **Plant** | **Mean no of days from sowing to anthesis** | | **Plant** | **Mean no of days from sowing to anthesis** | |
| --- | --- | --- | --- | --- | --- |
|  | **Control** | **Water stress** |  | **Control** | **Water stress** |
| Synthetic W7984 | 101 | 118 | SO_056 | 107 | 113 |
| Opata M85 | 98 | 104 | SO_057 | 115 | 92 |
| SO_001 | 111 | 121 | SO_058 | 113 | 110 |
| SO_002 | 104 | 98 | SO_059 | 111 | 95 |
| SO_003 | 109 | 97 | SO_060 | 111 | 96 |
| SO_004 | 104 | 93 | SO_061 | 109 | 116 |
| SO_005 | 107 | 128 | SO_062 | 104 | 96 |
| SO_006 | 107 | 122 | SO_063 | 120 | 110 |
| SO_007 | 112 | 96 | SO_064 | 107 | 101 |
| SO_008 | 104 | 99 | SO_065 | 111 | 95 |
| SO_009 | 118 | 99 | SO_066 | 114 | 109 |
| SO_010 | 114 | 92 | SO_067 | 110 | 94 |
| SO_011 | 101 | 118 | SO_068 | 124 | 124 |
| SO_012 | 103 | 95 | SO_069 | 109 | 125 |
| SO_014 | 104 | 95 | SO_071 | 116 | 100 |
| SO_015 | 113 | 137 | SO_072 | 112 | 96 |
| SO_016 | 105 | 126 | SO_073 | 117 | 96 |
| SO_017 | 108 | 122 | SO_074 | 108 | 100 |
| SO_018 | 104 | 96 | SO_075 | 125 | 101 |
| SO_019 | 109 | 99 | SO_076 | 114 | 101 |
| SO_020 | 107 | 120 | SO_077 | 112 | 110 |
| SO_021 | 109 | 104 | SO_078 | 118 | 114 |
| SO_022 | 115 | 98 | SO_079 | 124 | 114 |
| SO_023 | 106 | 103 | SO_080 | 116 | 130 |
| SO_024 | 119 | 122 | SO_081 | 119 | 128 |
| SO_025 | 112 | 97 | SO_082 | 114 | 151 |
| SO_026 | 113 | 105 | SO_083 | 116 | 116 |
| SO_029 | 116 | 97 | SO_084 | 124 | 118 |
| SO_030 | 107 | 103 | SO_085 | 118 | 102 |
| SO_031 | 108 | 131 | SO_086 | 119 | 101 |
| SO_032 | 125 | 93 | SO_088 | 119 | 125 |
| SO_033 | 107 | 104 | SO_089 | 126 | 144 |
| SO_034 | 118 | 97 | SO_090 | 122 | 121 |
| SO_035 | 112 | 125 | SO_091 | 103 | 99 |
| SO_036 | 117 | 123 | SO_092 | 114 | 92 |
| SO_037 | 119 | 143 | SO_093 | 113 | 97 |
| SO_038 | 113 | 115 | SO_094 | 115 | 100 |
| SO_039 | 115 | 128 | SO_095 | 122 | 116 |
| SO_040 | 121 | 128 | SO_096 | 111 | 107 |
| SO_041 | 117 | 95 | SO_097 | 108 | 92 |
| SO_042 | 123 | 107 | SO_098 | 112 | 99 |
| SO_043 | 116 | 102 | SO_099 | 116 | 99 |
| SO_044 | 111 | 99 | SO_100 | 126 | 128 |
| SO_045 | 110 | 107 | SO_101 | 119 | 91 |
| SO_046 | 113 | 130 | SO_102 | 116 | 95 |
| SO_047 | 112 | 96 | SO_103 | 117 | 107 |
| SO_048 | 117 | 103 | SO_104 | 111 | 105 |
| SO_049 | 107 | 104 | SO_106 | 111 | 106 |
| SO_050 | 115 | 116 | SO_110 | 112 | 95 |
| SO_051 | 116 | 112 | SO_111 | 125 | 128 |
| SO_052 | 119 | 93 | SO_112 | 112 | 145 |
| SO_053 | 124 | 96 | SO_113 | 115 | 110 |
| SO_054 | 125 | 117 | SO_114 | 114 | 131 |
| SO_055 | 121 | 118 |  |  |  |
