# Supplementary material for: Identification and validation of a major chromosome region for high grain number per spike under meiotic stage water stress in wheat (Triticum aestivum L.)
Source: PLoS One. 2018 Mar 8;13(3):e0194075. doi: 10.1371/journal.pone.0194075 (PMC5843344; doi:10.1371/journal.pone.0194075)
Supplement: S7 Table — (DOCX) [file pone.0194075.s007.docx]

S7 Table. Mean tiller number per plant for Synthetic W7984 parent, Opata M85 parent and 105 recombinant inbred lines (RILs) of Synthetic W7984×Opata M85 under both normal watering (control) and water stress during meiosis.

| **Plant** | **Mean tiller number per plant** | | **Plant** | **Mean tiller number per plant** | |
| --- | --- | --- | --- | --- | --- |
|  | **Control** | **Water stress** |  | **Control** | **Water stress** |
| Synthetic W7984 | 11 | 9 | SO_056 | 6 | 5 |
| Opata M85 | 9 | 8 | SO_057 | 5 | 5 |
| SO_001 | 8 | 5 | SO_058 | 5 | 7 |
| SO_002 | 7 | 9 | SO_059 | 7 | 8 |
| SO_003 | 8 | 7 | SO_060 | 6 | 7 |
| SO_004 | 6 | 8 | SO_061 | 6 | 6 |
| SO_005 | 6 | 5 | SO_062 | 6 | 8 |
| SO_006 | 5 | 7 | SO_063 | 6 | 6 |
| SO_007 | 10 | 12 | SO_064 | 7 | 9 |
| SO_008 | 5 | 6 | SO_065 | 7 | 8 |
| SO_009 | 6 | 9 | SO_066 | 8 | 9 |
| SO_010 | 9 | 8 | SO_067 | 7 | 9 |
| SO_011 | 4 | 9 | SO_068 | 8 | 5 |
| SO_012 | 6 | 10 | SO_069 | 7 | 3 |
| SO_014 | 9 | 8 | SO_071 | 6 | 9 |
| SO_015 | 11 | 8 | SO_072 | 8 | 11 |
| SO_016 | 10 | 7 | SO_073 | 8 | 7 |
| SO_017 | 7 | 7 | SO_074 | 9 | 7 |
| SO_018 | 8 | 10 | SO_075 | 10 | 6 |
| SO_019 | 8 | 5 | SO_076 | 7 | 9 |
| SO_020 | 8 | 6 | SO_077 | 8 | 7 |
| SO_021 | 7 | 8 | SO_078 | 6 | 5 |
| SO_022 | 9 | 8 | SO_079 | 6 | 4 |
| SO_023 | 8 | 10 | SO_080 | 6 | 8 |
| SO_024 | 8 | 7 | SO_081 | 6 | 6 |
| SO_025 | 8 | 9 | SO_082 | 7 | 4 |
| SO_026 | 8 | 8 | SO_083 | 8 | 8 |
| SO_029 | 8 | 7 | SO_084 | 7 | 5 |
| SO_030 | 7 | 6 | SO_085 | 7 | 7 |
| SO_031 | 6 | 7 | SO_086 | 6 | 4 |
| SO_032 | 7 | 8 | SO_088 | 6 | 7 |
| SO_033 | 7 | 10 | SO_089 | 8 | 7 |
| SO_034 | 8 | 9 | SO_090 | 8 | 8 |
| SO_035 | 9 | 4 | SO_091 | 10 | 12 |
| SO_036 | 6 | 6 | SO_092 | 6 | 9 |
| SO_037 | 8 | 4 | SO_093 | 8 | 7 |
| SO_038 | 8 | 5 | SO_094 | 7 | 10 |
| SO_039 | 10 | 7 | SO_095 | 7 | 8 |
| SO_040 | 10 | 7 | SO_096 | 6 | 6 |
| SO_041 | 7 | 8 | SO_097 | 9 | 11 |
| SO_042 | 9 | 5 | SO_098 | 8 | 8 |
| SO_043 | 6 | 10 | SO_099 | 7 | 8 |
| SO_044 | 8 | 10 | SO_100 | 5 | 6 |
| SO_045 | 9 | 8 | SO_101 | 7 | 7 |
| SO_046 | 6 | 6 | SO_102 | 8 | 11 |
| SO_047 | 8 | 5 | SO_103 | 7 | 5 |
| SO_048 | 5 | 6 | SO_104 | 7 | 6 |
| SO_049 | 7 | 8 | SO_106 | 9 | 5 |
| SO_050 | 7 | 6 | SO_110 | 6 | 10 |
| SO_051 | 7 | 7 | SO_111 | 8 | 6 |
| SO_052 | 6 | 5 | SO_112 | 5 | 6 |
| SO_053 | 9 | 8 | SO_113 | 4 | 4 |
| SO_054 | 8 | 8 | SO_114 | 6 | 4 |
| SO_055 | 8 | 6 |  |  |  |
