# Supplementary material for: Identification and validation of a major chromosome region for high grain number per spike under meiotic stage water stress in wheat (Triticum aestivum L.)
Source: PLoS One. 2018 Mar 8;13(3):e0194075. doi: 10.1371/journal.pone.0194075 (PMC5843344; doi:10.1371/journal.pone.0194075)
Supplement: S8 Table — (DOCX) [file pone.0194075.s008.docx]

S8 Table. Mean amount of water use (corresponding to the daily transpiration of the pots) which was determined by weighing the pots daily (kg) under water stress treatment during meiosis for Synthetic W7984 parent, Opata M85 parent and 105 recombinant inbred lines (RILs) of Synthetic W7984×Opata M85.

| **Plant** | **Water use during stress period** | **Plant** | **Water use during stress period** |
| --- | --- | --- | --- |
| Synthetic W7984 | 0.658 | SO_056 | 0.846 |
| Opata M85 | 0.822 | SO_057 | 0.539 |
| SO_001 | 0.804 | SO_058 | 0.828 |
| SO_002 | 0.559 | SO_059 | 0.746 |
| SO_003 | 0.720 | SO_060 | 0.625 |
| SO_004 | 0.778 | SO_061 | 0.659 |
| SO_005 | 0.886 | SO_062 | 0.726 |
| SO_006 | 1.013 | SO_063 | 0.710 |
| SO_007 | 0.772 | SO_064 | 0.742 |
| SO_008 | 0.639 | SO_065 | 0.716 |
| SO_009 | 0.621 | SO_066 | 0.865 |
| SO_010 | 0.779 | SO_067 | 0.753 |
| SO_011 | 0.801 | SO_068 | 0.803 |
| SO_012 | 0.682 | SO_069 | 0.877 |
| SO_014 | 0.769 | SO_071 | 0.765 |
| SO_015 | 0.835 | SO_072 | 0.696 |
| SO_016 | 0.726 | SO_073 | 0.650 |
| SO_017 | 0.720 | SO_074 | 0.709 |
| SO_018 | 0.805 | SO_075 | 0.740 |
| SO_019 | 0.806 | SO_076 | 0.722 |
| SO_020 | 0.765 | SO_077 | 0.774 |
| SO_021 | 0.728 | SO_078 | 0.792 |
| SO_022 | 0.673 | SO_079 | 0.492 |
| SO_023 | 0.809 | SO_080 | 0.763 |
| SO_024 | 0.745 | SO_081 | 0.805 |
| SO_025 | 0.736 | SO_082 | 0.856 |
| SO_026 | 0.777 | SO_083 | 0.769 |
| SO_029 | 0.728 | SO_084 | 0.850 |
| SO_030 | 0.643 | SO_085 | 0.689 |
| SO_031 | 0.768 | SO_086 | 0.780 |
| SO_032 | 0.758 | SO_088 | 0.846 |
| SO_033 | 0.744 | SO_089 | 0.797 |
| SO_034 | 0.675 | SO_090 | 0.797 |
| SO_035 | 0.742 | SO_091 | 0.752 |
| SO_036 | 0.732 | SO_092 | 0.754 |
| SO_037 | 0.773 | SO_093 | 0.693 |
| SO_038 | 0.779 | SO_094 | 0.686 |
| SO_039 | 0.838 | SO_095 | 0.788 |
| SO_040 | 0.807 | SO_096 | 0.849 |
| SO_041 | 0.744 | SO_097 | 0.803 |
| SO_042 | 0.748 | SO_098 | 0.778 |
| SO_043 | 0.785 | SO_099 | 0.641 |
| SO_044 | 0.778 | SO_100 | 0.771 |
| SO_045 | 0.735 | SO_101 | 0.650 |
| SO_046 | 0.823 | SO_102 | 0.724 |
| SO_047 | 0.735 | SO_103 | 0.645 |
| SO_048 | 0.701 | SO_104 | 0.643 |
| SO_049 | 0.650 | SO_106 | 0.770 |
| SO_050 | 0.763 | SO_110 | 0.749 |
| SO_051 | 1.756 | SO_111 | 0.823 |
| SO_052 | 0.481 | SO_112 | 0.967 |
| SO_053 | 0.758 | SO_113 | 0.796 |
| SO_054 | 0.761 | SO_114 | 0.787 |
| SO_055 | 0.951 |  |  |
